# Supplementary material for: Selective Agonism of Liver and Gut FXR Prevents Cholestasis and Intestinal Atrophy in Parenterally Fed Neonatal Pigs
Source: bioRxiv. 2024 Sep 7:2024.09.03.611073. Preprint. [Version 1] doi: 10.1101/2024.09.03.611073 (PMC11398320; doi:10.1101/2024.09.03.611073)
Supplement: Supplement 1 [file NIHPP2024.09.03.611073v1-supplement-1.pdf]

bioRxiv preprint doi: https://doi.org/10.1101/2024.09.03.611073; this version posted September 7, 2024. The copyright holder for this preprint (which was not certified by peer review) is the author/funder. This article is a US Government work. It is not subject to copyright under 17 USC 105 and is also made available for use under a CC0 license.

Supplementary Table 1. Forward and reverse primer sequences used for qRT-PCR.

Gene	Fwd	Rev
NR1H4	TTTGTGTCGTTTGCGGAGAG	GTTGCCCCCATTTTTACACTTG
NR0B2	GCCTACCTGAAAGGGACCAT	CAACGGGTGTCAAGCCTTTA
FGF19	CTGGGCCGCACGTGCACTAC	GGGCCCGTCTGAGTGGATGCG
ABCB11	TTTCATTCAGCGCCTGACCA	ACTCCAATGAGAGGGCTGAC
ABCB1	TATAACACCAGAGTGGGAG	GACAACCTTTTCACTTTCTG
ABCC4	CTTTGCCAATGCACTCCTTG	ACTCTTTGGATGCTGACGAC
SLC51A	TGTACAAGAACACTCGCTGC	GAACACACACACTATCGTGGG
SLC10A2	CTTTCGGAAACCTAAGGGACT	AAGAGCTTGCCCAGTGCAAAG
ABCG5	TCCAATGTGTGCCTTGAGTC	CCAGGATGACAAGAGTTGGG
ABCG8	GTCTGGCACCACCATCTACT	TCCACCCCATAGAAGTCAGC
CYP7A1	GAAAGAGAGACCACATCTCGG	GAATGGTGTTGGCTTGCGAT
CYP3A29	GTGGAGTGTTACATACGGGC	AGGTGATACTAGGTGGGGGT
CYP4A21	TTTTCCCGCTTGAGGAGTGC	ACTCGGTCTGTGTGTTGATGGA
CYP8B1	CCGGAAGAATATGTTGGAAT	AAGTCTAGTTTTCTCTTCGC
CYP27A1	ACTGAAGACCGCGATGAAAC	CAAAGGCGAATCAGGAAGGG
CYP7B1	AATACTTCCTCCCTTCTGCCC	GGGCAGAAGGGAGGAAGTATT
FABP6	CTCCTGCCTCATCCTTC	CATCGTAGTTCTTCTACTC
SLC10A2	ATAATGGGATGCTGTCCAGG	TAGATTAAGAGGCACAGCGG
ACTB	GGACCTGACCGACTACCTCA	GCGACGTAGCAGAGCTTCTC
